# Restoring Wnt/β-catenin signaling is a promising therapeutic strategy for Alzheimer’s disease

**DOI:** 10.1186/s13041-019-0525-5

**Published:** 2019-12-04

**Authors:** Lin Jia, Juan Piña-Crespo, Yonghe Li

**Affiliations:** 1 Department of Neuroscience, Mayo Clinic, Jacksonville, FL 32224 USA; 2 Fujian Provincial Key Laboratory of Neurodegenerative Disease and Aging Research, Institute of Neuroscience, Medical College, Xiamen University, Xiamen, 361102 China; 3 Neuroscience Initiative, Sanford Burnham Prebys Medical Discovery Institute, La Jolla, CA 92037 USA

**Keywords:** Wnt, Alzheimer’s disease, Neuronal survival, Neurogenesis, Synaptic plasticity, Drug target

## Abstract

Alzheimer’s disease (AD) is an aging-related neurological disorder characterized by synaptic loss and dementia. Wnt/β-catenin signaling is an essential signal transduction pathway that regulates numerous cellular processes including cell survival. In brain, Wnt/β-catenin signaling is not only crucial for neuronal survival and neurogenesis, but it plays important roles in regulating synaptic plasticity and blood-brain barrier integrity and function. Moreover, activation of Wnt/β-catenin signaling inhibits amyloid-β production and tau protein hyperphosphorylation in the brain. Critically, Wnt/β-catenin signaling is greatly suppressed in AD brain via multiple pathogenic mechanisms. As such, restoring Wnt/β-catenin signaling represents a unique opportunity for the rational design of novel AD therapies.

## Introduction

Alzheimer’s disease (AD) is the most common form of dementia accompanied by detrimental cognitive deficits and pathological accumulation of amyloid-β (Aβ) plaques and tau-containing neurofibrillary tangles [1]. As one of the most important medical and social problems, there is an urgent need for effective therapies. The amyloid hypothesis is based on neuropathological evidence showing Aβ aggregates (amyloid plaques) in AD brain and on the identification of over 200 mutations in the amyloid precursor protein (APP) and presenilin (PSEN) genes that cause familial AD (FAD) [1, 2]. The amyloid hypothesis has been the main driver of drug discovery efforts in the past 25 years; however, all clinical trials using anti-Aβ drugs as a treatment for AD have ended in failure [3]. Therefore, current paradigms in AD drug discovery have shifted to the development of drugs that target the multiple disease processes that support the progression of AD pathology, and novel targeted therapies are urgently needed to prevent and treat AD [3–5].

The Wnt/β-catenin signaling pathway is a significant pathway regulating cell proliferation, migration and differentiation, and Wnt proteins are key drivers of adult stem cells in mammals [6]. Studies have shown that dysregulated Wnt/β-catenin signaling plays an important role in the pathogenesis of AD [7]. In this review, we summarize our current understanding of regulation and function of the Wnt/β-catenin signaling pathway in AD brain and provide evidence indicating that the Wnt/β-catenin signaling pathway represents a new attractive therapeutic target for drug discovery in AD.

## Roles of Wnt/β-catenin signaling in physiological and pathophysiological processes in the brain

**Fig. 1:**
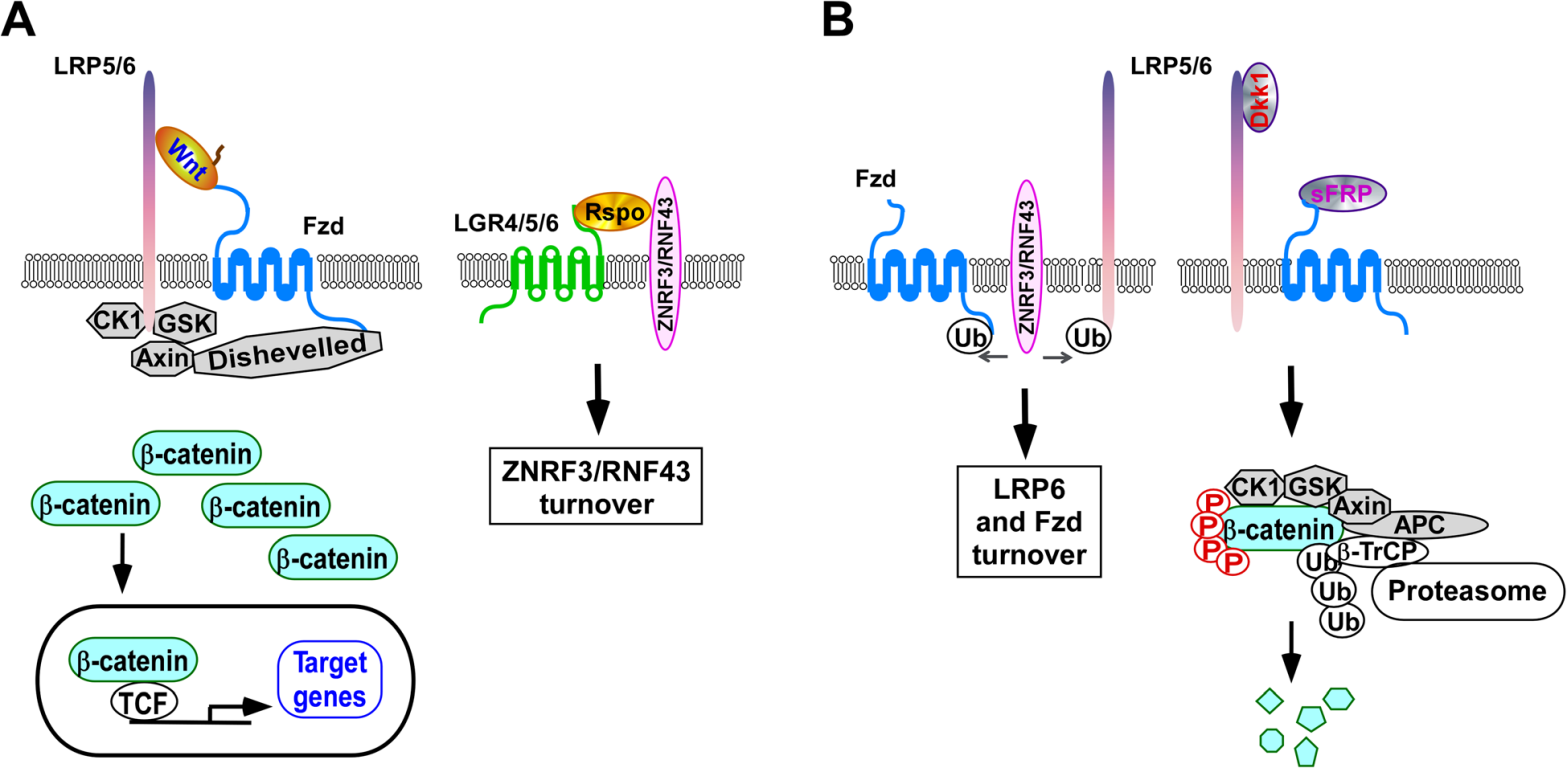
The Wnt/β-catenin signaling pathway. **a** When Wnt proteins bind to LRP5/6 and FZD, the phosphorylation and degradation of β-catenin is blocked, resulting in stabilization, accumulation and nuclear translocation of β-catenin and subsequent activation of the pathway. **b** When Wnt binding to receptors is blocked by Wnt antagonist Dkk1, SOST and sFRP, β-catenin is phosphorylated by Ck1 and GSK3β, and subsequently degraded by the 26S proteasome. Wnt receptor Fzd and Wnt co-receptor LRP5/6 are positively regulated by Rspo proteins and their receptors LGR4, LGR5 and LGR6, and negatively regulated by E3 ubiquitin ligases RNF43 and ZNRF3 at the cell surface

Wnt proteins are secreted glycoproteins that bind to the extracellular cysteine-rich domain of the Frizzled (Fzd) receptor family and Wnt co-receptor low density lipoprotein receptor-related protein 5 (LRP5) or LRP6 to activate the canonical Wnt/β-catenin signaling pathway. Binding of Wnt to the Fzd/LRP5/6 receptor complex results in inhibition of glycogen synthase kinase 3β (GSK3β) and stabilization of cytosolic β-catenin. Stabilized β-catenin then translocates into the nucleus, interacts with T-cell factor/lymphoid enhancing factor (TCF/LEF), and induces the expression of specific target genes (Fig. 1) [6]. Wnt/β-catenin signaling is tightly regulated at the cell surface by various secreted proteins and receptors. While Zinc and ring finger 3 (ZNRF3) and ring finger protein 43 (RNF43) promote LRP5/6 degradation [8–10], the extracellular molecule R-spondin (Rspo) together with its receptors leucine rich repeat containing G protein-coupled receptor 4/5/6 (LGR4/5/6) induces ZNRF3/RNF43 turnover, making LRP5/6 available on the cell surface for activation of the Wnt/β-catenin signaling pathway (Fig. 1) [11]. Moreover, Dickkopf (DKK) and soluble Frizzled-related protein (sFRP) bind to LRP5/6 and Fzd, respectively, and prevent LRP-Wnt-Fz complex formation in response to Wnts (Fig. 1 b) [6].

**Fig. 2:**
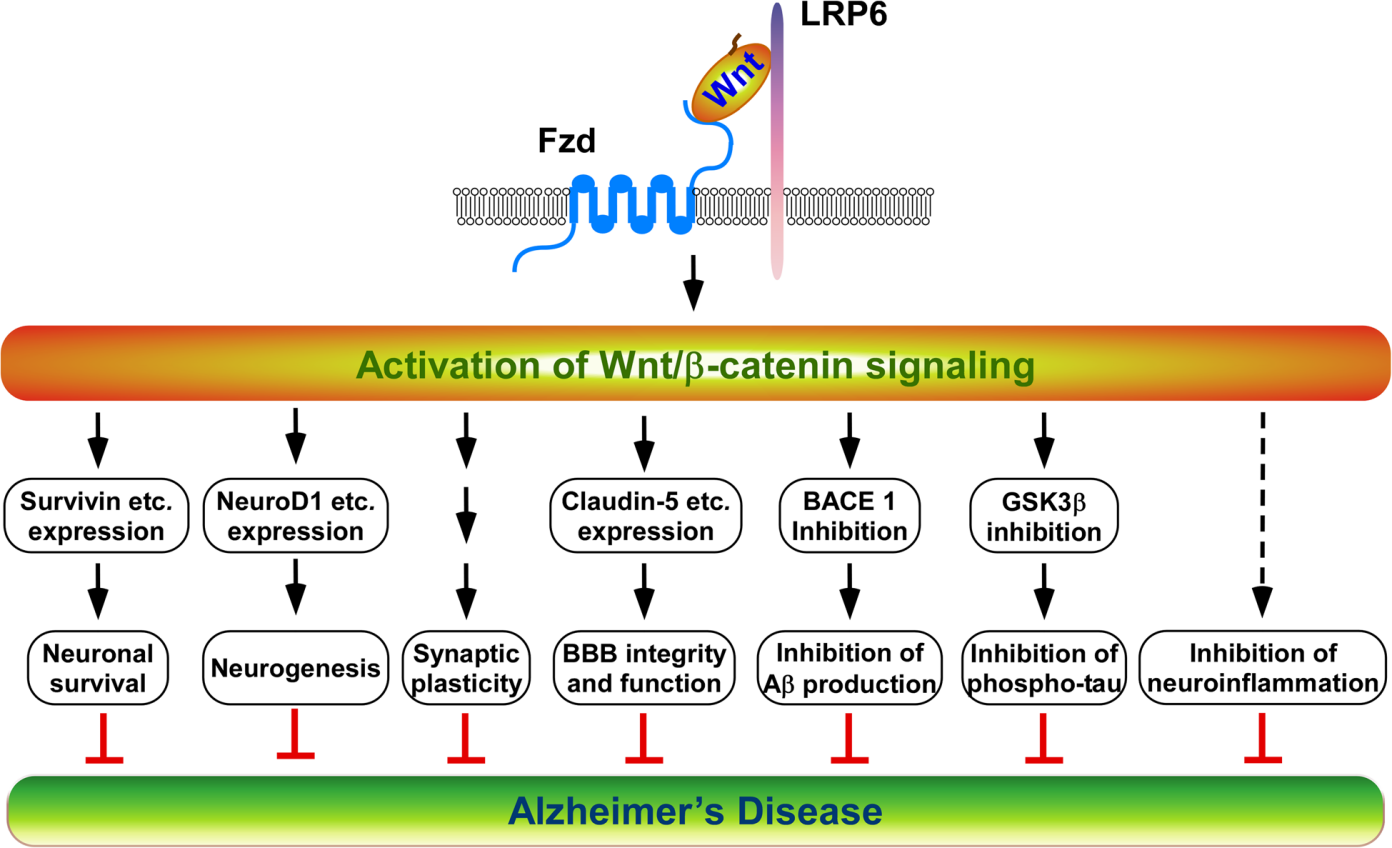
Restoring Wnt/β-catenin signaling is a promising therapeutic strategy for AD. Wnt/β-catenin signaling is able to regulate multiple different pathways in Alzheimer disease (AD) pathogenesis. Restoring Wnt/β-catenin signaling in the brain of the AD patients will enhance synaptic plasticity, neuronal survival, neurogenesis and BBB integrity and function and suppress Aβ production and tau phosphorylation. The role of Wnt/β-catenin signaling in neuroinflammation remains to be elucidated

In the rest of this section, we summarize our current understanding of the roles of Wnt/β-catenin signaling on multiple physiological and pathophysiological processes in AD brain (Fig. 2).

### Wnt/β-catenin signaling promotes neuronal survival and neurogenesis

The neurodegenerative process in AD is initially characterized by synaptic damage followed by neuronal loss [12]. The Wnt/β-catenin signaling pathway is a key pathway controlling cell death and survival [6]. Indeed, loss of Wnt/β-catenin signaling renders neuron more susceptible to Aβ-induced apoptosis [13], and activation of Wnt/β-catenin signaling rescues Aβ-induced neuronal death and behavioral deficits [14–17].

While there is a debate of the presence of neurogenesis in human adult brain [18], emerging evidence suggests that human hippocampus neurogenesis persists in aged adult brain and declines dramatically in AD brain [19–23]. Importantly, numerous studies have demonstrated that Wnt/β-catenin signaling is a key regulator of adult hippocampal neurogenesis [24–34]. Wnt7a plays a critical role in multiple steps of neurogenesis by activating Wnt/β-catenin signaling and specific downstream target genes involved in cell cycle control and neuronal differentiation [32]. Moreover, astrocyte-secreted Wnt proteins are decreased in aged mice, leading to suppression of Wnt/β-catenin signaling, down-regulation of survivin levels in neural progenitor cells (NPCs) and impaired adult neurogenesis during aging [29, 33]. Interestingly, neurogenesis induced by anti-aggregant tau mutant is associated with the activation of Wnt/β-catenin signaling [34]. Mechanistically, transcriptional activation of the mitotic regulator survivin, the basic helix-loop-helix transcription factor NeuroD1 and prospero-related homeodomain transcription factor Prox1, which all are essential for the generation of granule cells in the hippocampus, is dependent on activation of the Wnt/β-catenin signaling pathway [25, 26, 33, 35].

### Wnt/β-catenin signaling enhances synaptic plasticity

Synaptic plasticity is associated with higher brain functions such as learning and memory. Synapse loss, which occurs prior to neuronal death at early stages in AD brain, is a major correlate of cognitive impairment in AD brain [36, 37]. Recent studies have found that Wnt/β-catenin signaling is essential for synaptic plasticity [38, 39]. Wnt proteins are not only required for synapse formation, but they can modulate neurotransmission by acting both pre- and post-synaptically [38]. Long-term potentiation (LTP) is considered a cellular correlate of learning [40], and studies have demonstrated that Wnt proteins can promote LTP [41–44]. Significantly, neuronal activity can induce the release of several Wnt proteins such as Wnt1, Wnt2, Wnt3A and Wnt7a/b [41, 44–46] and decrease the expression of Wnt antagonist sFRP3 [28]; while LTP is severely impaired by functional blockade of endogenous Wnt proteins with Wnt antagonists DKK1 and SFRPs [43, 44, 47].

LRP6 is an essential Wnt co-receptor for activation of Wnt/β-catenin signaling on the cell surface. LRP6 is selectively localized to excitatory synapses, and is required for excitatory synapse development in vitro and in vivo [48]. Moreover, neuronal deficiency of LRP6 results in synaptic and cognitive abnormalities in aged mice [49]. All together, these studies indicate that neuronal LRP6-mediated Wnt/β-catenin signaling plays an important role in synaptic function and cognition.

DKK1 binds to LRP6 and blocks Wnt/β-catenin signaling on the cell surface. Mice with a dorsal hippocampal infusion of DKK1 exhibited impaired hippocampal-dependent novel object recognition memory with rapidly decreasing levels of key Wnt/β-catenin signaling proteins, including β-catenin, Cyclin D1, c-myc, Wnt7a, and PSD95 [50]. Induction of DKK1 expression in the hippocampus triggers synapse loss, synaptic dysfunction and memory impairment, all of which can be fully restored by reactivation of Wnt/β-catenin signaling after cessation of DKK1 expression in the hippocampus [43]. Collectively, these findings further demonstrate the critical role of LRP6-mediated Wnt/β-catenin signaling in synaptic plasticity.

### Wnt/β-catenin signaling is essential for the integrity and function of the blood-brain barrier (BBB)

The BBB protects the brain from exposure to neurotoxic blood-derived debris, cells and microbial pathogens. Therefore, BBB disruption allows influx of harmful substances into the brain, induces inflammatory and immune responses, and may subsequently initiate multiple pathways of neurodegeneration [51, 52]. BBB breakdown is an early biomarker of human cognitive impairment in AD [53]. It is observed before dementia, neurodegeneration and/or brain atrophy occur [54, 55]. BBB disruption is a key pathogenic feature of AD, which includes increased BBB permeability, microbleeding, diminished glucose transport, impaired Pgp-1 function (Aβ clearance), perivascular accumulation of neurotoxic blood-derived products, and cellular infiltration and degeneration of pericytes and endothelial cells [51, 53, 56]. Therefore, developing novel approaches that target BBB repair is a promising strategy for AD therapy.

In the past decade, studies have established that the Wnt/β-catenin pathway is a key pathway required not only for BBB formation but also for BBB integrity and function [57, 58]. By binding to Wnt receptor Fzd4 and Wnt co-receptor LRP5/6, Wnt ligands Wnt7a and Wnt7b, which are mainly produced by neurons and astrocytes in brain [59], activate Wnt/β-catenin signaling in BBB endothelial cells (ECs) [60–62], and activation of Wnt/β-catenin signaling is a key driver of BBB formation and function [60–62]. In addition, Reck, a GPI-anchored membrane protein, and Gpr124, an orphan GPCR, are essential cofactors on the cell surface for Wnt7a/Wnt7b-specific signaling in mammalian CNS angiogenesis, BBB integrity and function [63–68].

Brain ECs are held together by tight junctions, in which claudins are the main constituent. In addition, glucose transporter 1 (GLUT1), which is specifically expressed in BBB ECs, is responsible for the transport of glucose from the blood into the brain; and p-glycoprotein (Pgp-1) is an active efflux transporter highly expressed on the luminal surface of BBB endothelial cells. Mechanically, claudin-1, − 3 and − 5, the three major claudins expressed in brain ECs [69], are the transcriptional targets of Wnt/β-catenin signaling in BBB ECs [60, 67, 70]. Moreover, Wnt/β-catenin signaling drives the expression of the BBB-specific glucose transporter GLUT1 [61] and efflux transporter Pgp-1 in BBB ECs [71].

### Wnt/β-catenin signaling inhibits BACE1 expression and suppresses Aβ production/aggregation

One of the two major hallmarks of AD is the accumulation of amyloid plaques between neurons in the brain [72, 73]. Recent studies have found that Wnt/β-catenin signaling is able to inhibit amyloidogenic processing of APP by suppressing the transcription of the β-site APP cleaving enzyme (BACE1) [74, 75]. While activation of Wnt/β-catenin signaling reduces Aβ42 production and aggregation, Wnt inhibition induces opposite effects on APP processing and Aβ42 production/aggregation in a cellular model [75]. Moreover, loss of Wnt/β-catenin signaling induces AD-like neuropathological hallmarks in wild-type mice, and accelerates the development of AD-like pathology in an AD mouse model overexpressed human APP with two FAD mutations [76].

### Wnt/β-catenin signaling suppresses tau phosphorylation

Another major hallmark of AD is the presence of intracellular neurofibrillary tangles (NFTs) composed of hyperphosphorylated forms of the microtubule-associated protein tau (MAPT) in neurons [72, 73, 77]. GSK3β is an important kinase associated with hyperphosphorylation of tau protein (p-tau) at AD-relevant phosphorylation sites [78]. Activation of Wnt/β-catenin signaling results in the inhibition of GSK3β activity and subsequent suppression of tau phosphorylation. Indeed, the Wnt antagonist DKK1 is able to inhibit Wnt/β-catenin signaling and induce both tau hyperphosphorylation and neuronal death [79, 80]. In contrast, activation of Wnt/β-catenin signaling can inhibit Aβ-induced tau hyperphosphorylation and neuronal death [14, 17].

### Wnt/β-catenin signaling in microglia activity and neuroinflammation

Glia-mediated neuroinflammation is another pathological hallmark of AD [81–83]. Genetic factors such as rare variants of TREM2 (triggering receptor expressed on myeloid cells-2) strongly increase the risk of developing AD, confirming a role of neuroinflammation as a driving force in AD [84–88]. Interestingly, TREM2, which is exclusively expressed by microglia in brain, can promote microglial survival by activating Wnt/β-catenin signaling through posttranslational regulation of β-catenin [89]. On the other hand, Wnt antagonist sFRP1 and sFRP2 act as negative modulators of the disintegrin and metalloproteinase domain 10 protein (ADAM10) [90], which is an α-secretase responsible for shedding of the TREM2 ectodomain to produce soluble TREM2 (sTREM2) [86]; and recent studies indicate that sTREM2 displays a protective role in AD brain [91–93]. Moreover, activation of Wnt/β-catenin signaling with Wnt3a protein, LiCl, or TDZD-8 rescued microglia survival and microgliosis in *Trem2*^*−/−*^ microglia and *Trem2*^*−/−*^ mouse brain [89]. In addition, postnatal neuronal deletion of Wnt co-receptor LRP6 leads to microglial activation and neuroinflammation [49]. However, there are conflicting results regarding the roles of Wnt/β-catenin signaling on microglial activation and neuroinflammation [94]. Wnt/β-catenin signaling is active in microglia during neuroinflammation, raising the question as to whether enhanced Wnt/β-catenin signaling in microglia is harmful in AD brain [94], and further experimental work will be required to resolve this controversy.

## Wnt/β-catenin signaling is diminished in AD brain

While the Wnt/β-catenin signaling pathway is essential for brain function, this pathway is greatly suppressed via multiple pathogenic mechanisms in AD brain.

### Wnt/β-catenin signaling is down-regulated in the aging brain

It is well established that increasing age is the greatest risk factor for AD [95, 96]. Mounting evidence indicates a down-regulation of Wnt/β-catenin signaling in the aging brain, which may contribute to reduced neurogenesis and cognitive impairment [97]. In the aging brain, expression of Wnt proteins (such as Wnt 2, 3, 4, Wnt7b and Wnt10b) and disheveled (Dvl) proteins (such as Dvl2 and Dvl3) is down-regulated, while expression of Wnt antagonist DKK1 is up-regulated; leading to the suppression of Wnt/β-catenin signaling [29, 33, 98–100]. Importantly, the age-associated reduction in astrocytic levels of Wnt proteins impairs adult neurogenesis [29, 33], and rescue of secreted Wnt protein levels by exercise promotes adult neurogenesis [29].

### Dysregulation and malfunction of Wnt co-receptor LRP6 in AD brain

A growing body of evidence shows dysregulation and loss of function of Wnt co-receptor LRP6 contributes to down-regulation of Wnt/β-catenin signaling in AD. Firstly, two LRP6 SNPs and an alternatively splice variant that display impaired Wnt/β-catenin signaling activity, are associated with increased risk of developing AD [101, 102]. Secondly, expression of LRP6 is downregulated in AD brain [49], and deficiency in LRP6-mediated Wnt/β-catenin signaling contributes to synaptic dysfunction and amyloid pathology in AD [49]. Thirdly, apolipoprotein E4 (ApoE4), the most important risk factor for late-onset AD [103, 104], can inhibit Wnt/β-catenin signaling in neuronal LRP6-expressing PC-12 cells [105]. Finally, LRP6 physically interacts with APP and suppresses Aβ production [49, 106], while the Swedish familial AD variant of APP (APPSwe) displays reduced activation of Wnt/β-catenin signaling [106].

### Up-regulation of DKK1 expression results in suppression of Wnt/β-catenin signaling in AD brain

Aβ peptides can induce DKK1 expression and inhibit Wnt/β-catenin signaling in primary cortical neurons [80], and DKK1 expression in the adult hippocampus can induce synapse degeneration [43, 50]. Moreover, Aβ-induced synaptic loss can be attenuated by DKK1-neutralizing antibodies in mouse brain slices [107]. DKK1 is upregulated in postmortem AD brain, where it colocalizes with neurofibrillary tangles and distrophic neurites [80]. The upregulation DKK1 in AD brain and its colocalization with hyperphosphorylated tau have been also demonstrated in transgenic AD-like mouse models [108]. Critically, there is a pathogenic-positive feedback loop with Aβ stimulating DKK1 expression, thereby promoting synapse loss and driving further Aβ production [106].

### Activation of GSK3β in AD brain

The binding of Wnt protein to Fzd/LRP results in inhibition of GSK3β and consequent activation of Wnt/β-catenin signaling [6, 109]. GSK3β is one of two major kinases responsible for β-catenin phosphorylation, and activation of GSK3β induces β-catenin phosphorylation and degradation [110]. The increased activity of GSK3β has been found in the brain of AD patients [111, 112], which could be resulted from the up-regulation of DKK1 and down-regulation of LRP6 in the AD brain. A recent study shows that a significant decrease in β-catenin protein levels is inversely associated with increased activation of GSK3β in the prefrontal cortical lobe structures of human AD brains [113], further strengthening the notion that GSK3β activity is associated with Wnt/β-catenin signaling in AD brain. Notably, GSK3β is a key kinase for tau phosphorylation, and overactivation of GSK3β is intimately linked to tau hyperphosphorylation, Aβ deposition, plaque-associated microglial-mediated inflammatory responses and memory impairment [111, 112, 114].

### AD-associated APP mutants suppress Wnt/β-catenin signaling in AD brain

APP mutations can cause early-onset FAD [115, 116]. While studies using wild-type APP produced conflicting results regarding the activity of Wnt/β-catenin signaling, studies with FAD-associated APP mutants consistently revealed that Wnt/β-catenin signaling is inhibited by FAD-associated APP mutants [106, 117]. Studies in APP transgenic and knockout animal models and human AD brains demonstrated that APP and β-catenin co-localize and form a physical complex that is not present in healthy controls [118], and that β-catenin expression is greatly increased in hippocampal CA1 pyramidal cells from APP knockout mice [117]. Studies in primary neurons showed that overexpression of APP and its mutants promoted β-catenin degradation, while APP knockdown produced opposite effects [117].

### Regulation of Wnt/β-catenin signaling by PSEN1 and its AD-associated mutants in AD brain

Mutations in *PSEN1* are among the major causes of early-onset FAD [116, 119]. In the hippocampus, PSEN1 and PSEN2 play an important role in the regulation of synaptic plasticity, Aβ production and intracellular Ca^2+^ homeostasis [120, 121]. Many studies support the notion that PSEN1 and its mutants associated with FAD are negative regulators of Wnt/β-catenin signaling [13, 122–128], although inconsistent results with respect to the effects of FAD-associated PSEN1 mutants on Wnt/β-catenin signaling have been reported [129]. In a genetic modifier screening, *Drosophila* PSEN was identified as a suppressor of wingless/Wnt signaling [125]. PSEN deficiency enhances Wnt/β-catenin signaling through relocalization of GSK3 to the late-endosomal compartment [130], and facilitates the stepwise phosphorylation of β-catenin independently of the Wnt-controlled Axin complex [126]. Moreover, the expression of β-catenin is reduced in AD patients carrying *PSEN1* mutations [13], and *PSEN1* mutations associated with AD cause a perturbation in the intracellular trafficking of β-catenin [122], decrease the stability and/or enhance the degradation of β-catenin [123, 124]. However, some FAD-associated PSEN1 mutants such as FAD-PSEN1^L286V^ and -PSEN1^M146L^ fail to induce β-catenin degradation [62, 131, 132]. Instead, FAD-PSEN1^L286V^ can upregulate a subset of TCF/β-catenin transcription by enhancing the level of cAMP-response element-binding protein (CREB)-binding protein (CBP) [131].

## Targeting Wnt/β-catenin signaling in AD therapy

Giving that the Wnt/β-catenin pathway is greatly suppressed in the brain of AD patients, restoring Wnt/β-catenin signaling represents a unique opportunity for rational AD therapy (Fig. 2).

### The active lifestyle-induced cognitive improvement is associated with activation of Wnt/β-catenin signaling

A physically active lifestyle in adults and the elderly can improve brain health and reduce cognitive impairment associated with aging [133]. It has been reported that the enhancement of cognitive function by lifelong exercise is associated with induction of Wnt gene expression in the hippocampus [134]. Particularly, long-term moderate exercise and environmental enrichment can stimulate Wnt/β-catenin signaling by reducing DKK1 protein levels and increasing LRP6 and Wnt3a protein levels in hippocampus of adult animals [29, 135]. These findings suggest that activation of Wnt/β-catenin signaling is a potential mechanism underlying the cognitive improvement associated with an active lifestyle.

### Estrogen-induced neuroprotection is associated with inhibition of DKK1 expression

Estrogens can exert numerous protective actions in the adult brain, and reduced estrogen levels in adulthood are associated with increased risk of AD in women [136, 137]. In female rats, long-term estrogen deprivation leads to elevation of basal DKK1 expression and suppression of Wnt/β-catenin signaling in the CA1 hippocampal region [138]. Moreover, estrogen-induced neuroprotection and attenuation of tau phosphorylation are associated with DKK1 inhibition and subsequent activation of Wnt/β-catenin signaling [139]. Together, these findings suggest that inhibition of DKK1 is a potential mechanism for estrogen-induced neuroprotection.

### GSK3β inhibitors

The activity of GSK3β is negatively regulated by Wnt/β-catenin signaling [6, 109]. Given the key role of GSK3 activity on the pathogenesis of AD, various GSK3β inhibitors have been shown to inhibit tau hyperphosphorylation and reduce Aβ levels in both neuronal and nonneuronal cells, and rescue cognitive deficits in several murine models of AD [112, 140]. However, due to the wide range of GSK3β substrates and physiological actions, the use of GSK3β inhibitors in clinical studies in AD patients has been disappointing [112, 141]. Therefore, novel GSK3β inhibitors that selectively regulate the activity of this kinase in Wnt/β-catenin signaling in brain are highly desirable.

### DKK1 inhibitors

Suppression of Wnt/β-catenin signaling by Aβ-induced up-regulation of DKK1 expression in AD brain suggests DKK1 inhibition is a potential therapeutic strategy for restoring Wnt/β-catenin signaling in AD [142]. Indeed, it has been found that DKK1 anti-sense oligonucleotides (ASO) attenuate neuronal apoptosis and prevent tau hyperphosphorylation in Aβ-treated neurons [80], and that DKK1-neutralizing antibodies attenuate synapse loss induced by Aβ in mouse brain slices [107].

A virtual screen of the National Cancer Institute database for chemical compounds identified a small molecule, IIIC3 (NCI8642, gallocyanine), as a DKK1 inhibitor [143]. IIIC3 can inhibit DKK1 binding to LRP6 with an IC_50_ of 3 μM [143], and revert DKK1-mediated inhibition of Wnt/β-catenin signalling [143, 144]. Moreover, IIIC3 can reduce basal blood-glucose concentrations and improve glucose tolerance in mice [143]. Interestingly, IIIC3 and its derivatives can decrease DKK1-induced Tau phosphorylation [145, 146]. However, it is unclear whether these gallocyanine inhibitors of DKK1 can cross the BBB.

### Other activators of Wnt/β-catenin signaling

WASP-1 is a small molecule Wnt activator with an EC_50_ of about 250 nM in the Wnt reporter assays [147]. Although the exact mechanism of action of this compound is unclear, activation of Wnt/β-catenin signaling by bilateral intra-hippocampal infusion of WASP-1 rescues memory loss and improves synaptic dysfunction in murine models of AD [148, 149].

Curcumin, a natural compound found in the plant turmeric (*Curcuma longa*), displays protective effects in various animal models of AD [150, 151]. Studies have shown that curcumin can potentially promote Wnt/β-catenin signaling by increasing the expression of Wnt proteins and Wnt co-receptor LRP5/6 and suppressing the expression of Wnt antagonist DKK1 [152, 153]. However, because of its poor brain bioavailability, curcumin is of limited use in human AD patients, and there is currently lack of clinical evidence to support its therapeutic use in AD patients [150, 151]. Recently, it has been reported that curcumin nanoparticles, which exhibit increased brain bioavailability, potently stimulate adult neurogenesis and mitigate cognitive impairment in the AD model via activation of the Wnt/β-catenin pathway [153].

Statins are a class of drugs typically used to lower blood levels of cholesterol by reducing the production of cholesterol by the liver, and many studies suggest that statin use might protect against AD pathology [154–158]. Several studies have shown that statins are activators of Wnt/β-catenin signaling [159–164]. Mechanistically, statins enhance Wnt/β-catenin signaling through regulation of isoprenoid synthesis, which is not associated with cholesterol levels [163]. Interestingly, it has been demonstrated that lovastatin protects neuronal cells from Aβ-induced apoptosis via activation of Wnt/β-catenin signaling [159], and that simvastatin suppresses neural cell apoptosis and enhances locomotor recovery by stimulating Wnt/β-catenin signaling after spinal cord injury [164]. Moreover, simvastatin can promote Wnt/β-catenin signaling in the hippocampus of adult mice, and enhance neurogenesis both in cultured adult neural stem cells and the mouse hippocampus [163]. All together, these findings suggest that activation of Wnt/β-catenin signaling is one of the mechanisms by which statins are beneficial in AD and other neurological disorders.

## Conclusion and perspectives

Compared to a large number of Wnt inhibitors as potential agents for cancer prevention and treatment, there are only a few Wnt activators reported in the literature [6, 165]. Particularly, there are no specific BBB-permeant Wnt activators that can be used as potential candidates for the treatment of AD or other neurological disorders. While Wnt/β-catenin signaling is critical for synaptic plasticity, neuronal survival, neurogenesis and many other brain functions, it is greatly diminished in the brain of AD patients. Therefore, small molecule Wnt activators that restore Wnt/β-catenin signaling in brain, particularly those targeting Wnt antagonist DKK1, Wnt receptor LRP6 and tau regulator GSK3β, could represent novel therapeutic tools for the treatment for AD. In addition, emerging evidence indicates that Wnt/β-catenin signaling is also disrupted in other neurodegenerative disorders such as Parkinson’s disease [166–171]. Thus, Wnt activators hold a great therapeutic potential for other neurological disorders.

It is well established that Wnt/β-catenin signaling plays a key role in the regulation of bone mineral density, and that the Wnt/β-catenin signaling pathway is an attractive target for therapeutic intervention to restore bone strength in patients with osteoporosis disorders [172, 173]. Interestingly, AD patients have a much greater risk of suffering osteoporosis [174–177]. In addition, low bone mineral density phenotypes are manifested in AD mouse models [178–181]. Particularly, a recent study demonstrated that Wnt/β-catenin signaling is disrupted both in brain and bone of the htau mouse model of tauopathy, which has an early low bone mineral density phenotype [179]. Therefore, osteoporosis and AD could share a key mechanism of pathogenesis [182], and Wnt activators might not only reduce cognitive impairment but also prevent bone loss in the AD patients.

There is always a concern that overstimulation of Wnt/β-catenin signaling can promote cancer because aberrant activation of Wnt/β-catenin signaling can lead to tumor formation [6, 109]. However, there are no reports of increased incidence of cancer in families carrying *LRP5* gain-of-function mutations, and *Sost*- or *Dkk1*-deficient animals do not have an increased risk of tumor developments [183]. Nevertheless, the therapeutic application of Wnt activators should be given precisely to restore, but not overactivate, the Wnt/β-catenin signaling pathway in AD patients.

## Data Availability

Not applicable.

## Abbreviations

AD: Alzheimer’s disease
ADAM10: The disintegrin and metalloproteinase domain 10 protein
ApoE4: Apolipoprotein E4
APP: Amyloid precursor protein
ASO: Anti-sense oligonucleotides
BACE1: The β-site APP cleaving enzyme
BBB: Blood-brain barrier
CBP: CAMP-response element-binding protein (CREB)-binding protein
DKK: Dickkopf
Dvl: Disheveled
ECs: Endothelial cells
FAD: Familial Alzheimer’s disease
Fzd: Frizzled
GLUT1: Glucose transporter 1
GSK3β: Glycogen synthase kinase 3β
LGR: G protein-coupled receptor
LRP: Low density lipoprotein receptor-related protein
LTP: Long-term potentiation
MAPT: The microtubule-associated protein tau
NFTs: Intracellular neurofibrillary tangles
NPCs: Neural progenitor cells
Pgp-1: P-glycoprotein
PSEN: Presenilin; Rspo: R-spondin
RNF43: Ring finger protein 43
sFRP: Soluble Frizzled-related protein
sTREM2: Soluble TREM2 TCF/LEF: T-cell factor/lymphoid enhancing factor
TREM2: Triggering receptor expressed on myeloid cells-2
ZNRF3: Zinc and ring finger 3
